# Youth Judokas Competing in Higher Age Groups Leads to a Short-Term Success

**DOI:** 10.3390/children9111737

**Published:** 2022-11-11

**Authors:** Jozef Simenko

**Affiliations:** School of Life and Medical Sciences, University of Hertfordshire, Hatfield AL10 9EU, UK; j.simenko@herts.ac.uk

**Keywords:** combat sports, dropout, experience, performance, playing-up, periodisation

## Abstract

Coaches of youth judo athletes might be under the influence of some extraordinary elite judo athletes that have won elite competitions at a relatively young age and might put youth athletes under pressure to gain as much fighting experience as fast as possible. The present study aims to present a 5-year competition structure, volume and age competition categories (ACC) range in which youth judokas competed with 10-year dropout status. Data from 46 judokas were collected (M = 24; F = 22) for four categorisation classes (National-NC; Perspective-PC; International-IC; World class-WC). Competitive structure, volume, performance and number of ACC were collected from 2009 to 2013 for all age groups from scores and standings records of the National Federation. Youth judokas competed in 8 (±2) competitions per year and also competed in 3 (±1) ACC. Abroad competitions affect the fighting experience and competitive success (CS). CS showed positive correlations with the number of ACC in the year 2009 (*p* = 0.01), 2010 (*p* = 0.01) and 2011 (*p* = 0.04). The final observed years’ CS 2012 (*p* = 0.009) and 2013 (*p* = 0.002) showed a negative association with the number of ACC. CS in the final observed year 2013 showed a positive association (*p* = 0.012) with the dropout status in 2018 and a negative one with the number of abroad competitions in 2013 (*p* = 0.029). In total, 52% dropout was noted in 10 years. This ‘’playing-up’’ approach was shown to be successful in creating youth medalists. However, just in the short term, if implemented for too long, it starts to affect competition success negatively and increases youth athletes’ dropout. Therefore, coaches should include more competitions abroad in competitors’ primary age group, while training sessions could be done with higher age groups which would allow for gathering additional experience in a more controlled environment in their yearly periodisation.

## 1. Introduction

Participation in youth sports is a widely known phenomenon, and through it, youth athletes can increase their physical activity and develop physical, psychological and social skills [1,2]. During the late phases of adolescence or during the beginning of adulthood, we can see the development of technical-tactical [3] and some physiological variables, such as anaerobic capacity [4] and strength [5]. At that time, involvement in elite youth sports is slowly starting to emerge. It was highlighted that recently youth athletes had begun early specialisation in their selected sport at increasingly younger ages [6,7] which might be the consequence of increased pressure from coaches and/or parents on them to achieve top results [8,9]. However, early specialisation is not recommended [7] due to concerns about the potential for injury [10,11] and the development of psychological burnout against a host of other potential sociological issues. Moreover, early specialisation’s harmful effects have provoked the American Orthopedic Society for Sports Medicine (AOSSM) to publish an Early Sport Specialisation Consensus Statement [12]. Additionally, the struggle for competitive standings during childhood and youth with highly specialised training has been highly criticised [13,14].

In judo, competitions at the elite youth level were extended in 2009 with the introduction of the World Judo Cadet Championship (WCC) [15]. Additionally, a year later, in 2010, another high-level youth competition was introduced in the form of the Youth Olympic Games (YOG) [16], which could be one of the reasons for the added pressure on high standings at an early age. The consequence of these competitions will be seen in additional attention directed to the specialised development of athletes from 13 to 14 and 15 to 16-year-old groups [17]. Coaches in judo might be under the influence of some extraordinary elite judo athletes that have won gold medals in Senior World Championships and Olympic games at a relatively young age. Like the case of Kye Sun-Hui winning Olympic gold at the age of 16, Ilias Iliadis winning a gold Olympic medal at the age of 17, Teddy Riner, winning the World Senior Championship at the age of 18 and recently the youngest senior World champion Daria Bilodid winning the Senior World championship at the age of 17. Additionally, research shows that Japan, as one of the most successful judo countries in the world, starts the specialisation in female judokas at an average age of 14.2 years, while their male athletes start at an average age of 16.3 years [15]. Research has shown that an essential predictor of success in elite youth judokas of lower weight categories (LWC) is speed, while in LWC females, specific endurance and peak power bench press were identified as more important predictors [18,19]. In heavier weight categories for males and females, overall maximum strength and hand grip strength with body height and arm span was identified as the most vital predictor of success [18,19]. Additionally, factors like technique, tactical–cognitive skills, and visual tracking were highlighted to be essential factors in successful judo performance [20,21]. In youth judo athletes, somatic growth and years of formal training have also contributed to the increased neuromuscular performance of the upper and lower limbs [22], which are essential for a successful contest. 

The theory of Ericsson’s model or the Theory of Deliberate practice (TODP) [23] highlights the 10,000 h of deliberate practice to achieve elite performance (across various domains, including sports). Presuming the youth athlete starts training judo at the age of 6–9 years old; with training sessions of youth lasting from 60 to 90 min per session and conducted three times per week for the first 3–4 years (936 h in 4 years) and afterwards 4–5 times per week lasting for 90 to 120 min per session (416 h in 1 year), athletes would achieve this number in an average of 25 years. However, this theory and timeline are not in line in the eyes of coaches training their youth judokas to achieve good results in YOG and Cadet World Championships. Additionally, from a critical point of view, early specialisation would be inevitable if the TODP were completely applied to sports practice. Additional criticism was noted in the literature that TODP does not consider early development experiences, creativity developed via play and practice, or the impact of hereditary and environmental factors [24]. It was demonstrated that expert performance could also be achieved with 3000 to 4000 h of sport-specific training in sports where athletes’ peak is achieved after the age of 20 years [25]. 

The tendency is to train and compete more, consequently getting more experience, a good youth result and elite youth performance. However, the literature has shown that successful competitive performance in early judo competition was not associated with success later in adulthood, as only 7% of the male and 5% of the female athletes had maintained their competitive levels [17]. However, the theory and previous research have been challenged by the latest Tokyo 2020 Olympic games, where 41 medalists won medals at the Cadet and/or Junior World Championships, while 12 were medalists in WCC [26,27]. On the other side, high competitive pressure on youth athletes can cause a high dropout as research tracking a 5-year (2017–2021) youth cadet judokas competitive results showed that 70.6% of those youth judokas had finished their sporting carrier [28]. This could also be because judo possesses two High-risk burnout characteristics (1) involved in technical and/or (2) weight-dependent sports [29]. The current status in elite youth sports is concerning as it was reported that about every tenth young elite athlete reported burnout or depressive symptoms of potential clinical relevance [30]. Additionally, lack of time, fatigue and possible entry to the university were also identified as important factors for dropping out of the sport [31].

Therefore, knowing the practices of youth athletes and their yearly competition volume and how this changes as they get older and how they gain competitive experience is of great importance for researchers and coaches. However, there is a lack of studies that have focused on youth judokas competition volume, their structure of home and abroad competitions and competition range regarding youth judokas competing in higher age categories to gain competition experience. Therefore, this study aims to retrospectively analyse and present The present study aims to present a 5-year competition structure, volume and age competition categories (ACC) range in which youth judokas competed with 10-year dropout status. 

## 2. Materials and Methods

This is a retrospective study that collected the competition data from the freely accessible web page of the Slovenian Judo Federation (SJF) and its history of competition backing to the year 2009 (https://judoslo.si/ranking/team/all/2009, accessed on 5 May 2021). Therefore, the year 2009 was selected as a starting year. Afterwards, the competition list was cross-referenced with the list of categorised judo athletes of the Olympic Committee of Slovenia (OCS) for 2009 (https://www.olympic.si/evidenca, accessed on 7 May 2021). The age of birth criterion for inclusion in the analysis was 1990 and younger. The year 1990 was selected as a cut of age because the athletes would have been at a maximum of 19 years old in 2009 and their main age competition category would be juniors-U21. Additionally, from older competitors, we would only be able to observe the competition structure and volume from the senior and/or U23 category. The OCS categorisation levels from which we selected the competitors were: National, Perspective, International and World class sports categorisation level. 

### 2.1. Participants

Data from 46 judokas (Male = 24; Female = 22) were collected. The national class presented 15 judokas (Male = 11, Female = 4), the Perspective class 23 judokas (Male = 11, Female = 12), the International class 5 judokas (Male = 2, Female = 3) and the World class 3 judokas (Female = 3). The records of the Slovenian Judo cup for the years from 2009 to 2013 in all age groups they were competing were used to gather the following variables: Competitive structure and volume via a number of home, abroad and the total number of competitions; Competitive performance as final points in the Cup standing [32]. According to athletes’ age in 2009, a primary age competition category was determined and afterwards, all higher age categories were checked from the records. Youth athletes were competing in age competition categories: U12, U14, U16, U18, U21, U23 and seniors. The 10-year dropout was identified by checking the database to see if an athlete competed in 2018. Additionally, the years 2019 and 2020 were also checked. That was done to be sure the athlete was not out in 2018 because of possible injury or other factors and was back competing the following year. 

No ethical issues were present in analysing these data as they were obtained in secondary form and not generated by experimentation via open-access websites, and athletes’ personal information was not reported [33,34,35]. Therefore, written informed consent and ethical institutional approval were not needed.

### 2.2. Statistical Analysis

Data were processed and presented using the SPSS for Windows 28.0 statistical package (SPSS, Inc., Chicago, IL, USA) and descriptive statistics were used. The Shapiro–Wilk test was used to assess the normality of the data. To determine correlations between selected variables, Spearman correlation coefficients was used. The non-parametric Kruskal–Wallis Test was performed to compare selected variables across the 4 categorisation levels with post hoc paired comparisons by a Mann–Whitney U test with Bonferroni adjustment. Additionally, the eta-squared effect size values were calculated: 0.01—small, 0.06—medium, 0.14—large [36]. Statistical significance was set at *p* ≤ 0.05.

## 3. Results

Characteristics of the sample are presented in Table 1. The mean age of the judokas was 19.45 (±2.41) years, with 47.8% male and 52.2% female representatives. Starting age category of participants in 2009 was 5.15 (±1.55), meaning that the sample’s primary age competition category was U16. The primary age competition category in the year 2009 for the National class was between U14 and U16 (5.53 ± 1.36), for the Perspective class was between U14 and U16 (5.61 ± 1.37), for the International class was U21 (3.2 ± 0.45) and for the World class was U21 (3 ± 0).

**Figure 1:**
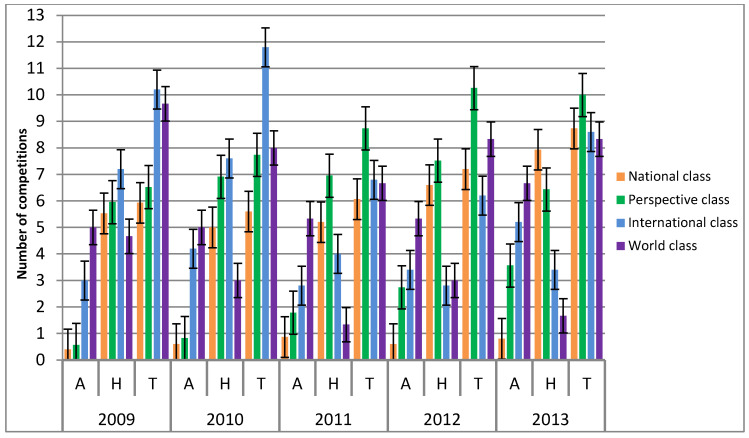
The distribution of abroad (A), home (H) and total number of competitions (T) of different categorisation classes from years 2009 to 2013.

Figure 1 presents the distribution of abroad (A), home (H) and the total number of competitions (T) of National, Perspective, International and World class categorised judokas from years 2009 to 2013. The National Class (NC) categorised judokas competition structure was: the year 2009 (A 0 ± 1; H 6 ± 1; T 6 ± 4); the year 2010 (A 1 ± 1; H 5 ± 1; T 6 ± 4); the year 2011 (A 1 ± 2; H 5 ± 2; T 6 ± 3); the year 2012 (A 1 ± 1; H 7 ± 3; T 7 ± 3) and in the year 2013 (A 1 ± 1; H 8 ± 4; T 9 ± 4). The Perspective Class (PC) judokas competition structure was: the year 2009 (A 1 ± 1; H 6 ± 3; T 7 ± 4); the year 2010 (A 1 ± 2; H 7 ± 3; T 8 ± 4); the year 2011 (A 2 ± 2; H 7 ± 3; T 9 ± 4); the year 2012 (A 3 ± 3; H 8 ± 3; T 10 ± 4) and in the year 2013 (A 4 ± 2; H 6 ± 4; T 10 ± 5). The International Class (IC) judokas competition structure was: the year 2009 (A 3 ± 2; H 7 ± 2; T 10 ± 1); the year 2010 (A 4 ± 2; H 8 ± 2; T 12 ± 4); the year 2011 (A 3 ± 1; H 4 ± 1; T 7 ± 2); the year 2012 (A 3 ± 2; H 3 ± 1; T 6 ± 3) and in the year 2013 (A 5 ± 2; H 3 ± 2; T 9 ± 1). The World Class (WC) judokas competition structure was: the year 2009 (A 5 ± 1; H 5 ± 1; T 10 ± 1); the year 2010 (A 5 ± 4; H 3 ± 1; T 8 ± 5); the year 2011 (A 5 ± 5; H 1 ± 1; T 7 ± 6); the year 2012 (A 5 ± 1; H 3 ± 2; T 8 ± 1) and in the year 2013 (A 7 ± 2; H 2 ± 1; T 8 ± 2). 

**Figure 2:**
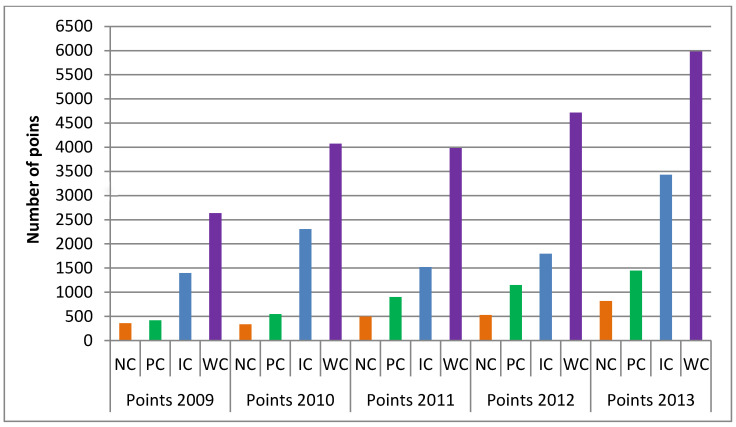
Competition success (CS) as total achieved points from scores and standings from all age competition categories from years 2009 to 2013 for different categorisation classes. NC—National class, PC—Perspective class, IC—International Class, WC—World class.

Figure 2 presents the distribution of total achieved points from standings from all age competition categories from 2009 to 2013 for different categorisation classes. The NC judokas achieved the following CS: 2009/361 ± 589 points; 2010/339 ± 396 points; 2011/498 ± 549 points; 2012/529 ± 350 points and in the year 2013/819 ± 502 points. The PC judokas achieved the following CS: 2009/422 ± 528 points; 2010/547 ± 603 points; 2011/900 ± 783 points; 2012/1152 ± 772 points and in the year 2013/1447 ± 962 points. The IC judokas achieved the following CS: 2009/1396 ± 637 points; 2010/2306 ± 1000 points; 2011/1522 ± 473 points; 2012/1797 ± 1392 points and in the year 2013/3428 ± 434 points. The WC judokas achieved the following CS: 2009/2637 ± 946 points; 2010/4076 ± 2749 points; 2011/3982 ± 3456 points; 2012/4717 ± 1848 points and in the year 2013/5980 ± 551 points. In a 5-year period judokas in average accumulated: NC judokas 2545 ± 1832 points, PC judokas 4467 ± 2770 points, IC judokas 10,449 ± 1948 points and WC judokas 21,392 ± 5102 points.

**Figure 3:**
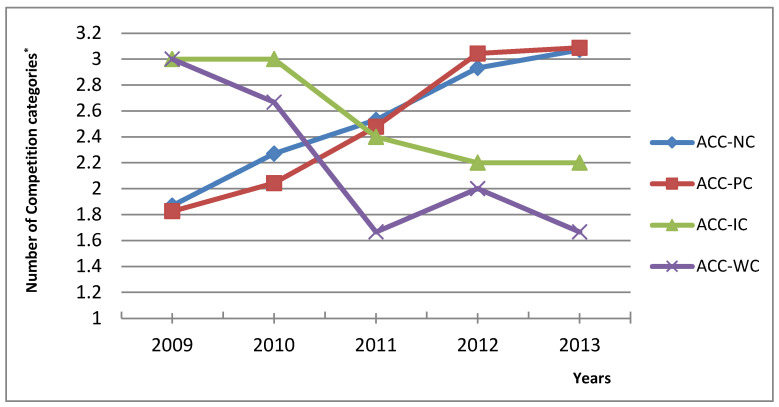
Display of competitions in higher age categories besides judokas primary age category. * 1 = Primary age category; 1 < number of higher age categories. ACC—average number of competitions categories; NC—National class; PC—Perspective class; IC-International Class; WC—World class.

Figure 3 presents how many higher age categories on top of their primary age category (AC) were judokas competing in the years 2009 to 2013. NC judokas competed: 2009/2 ± 1 AC; 2010/2 ± 1 AC; 2011/3 ± 1 AC; 2012/3 ± 1 AC and 2013/3 ± 1AC. PC judokas competed: 2009/2 ± 1 AC; 2010/2 ± 1 AC; 2011/3 ± 1 AC; 2012/3 ± 1 AC and 2013/3 ± 1AC. IC judokas competed: 2009/3 ± 1 AC; 2010/3 ± 0 AC; 2011/2 ± 1 AC; 2012/2 ± 1 AC and 2013/2 ± 1 AC. WC judokas competed: 2009/3 ± 0 AC; 2010/3 ± 1 AC; 2011/2 ± 1 AC; 2012/2 ± 0 AC and 2013/2 ± 0 AC. 

**Figure 4:**
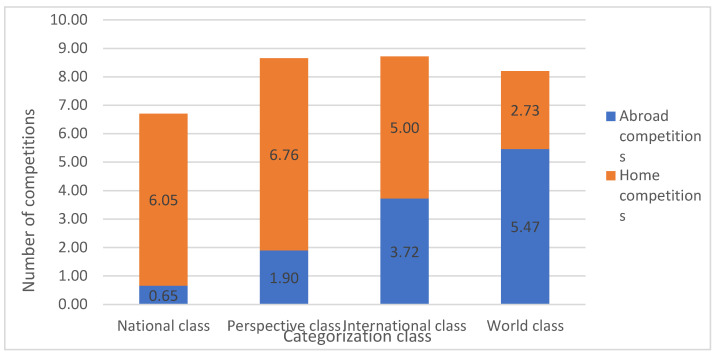
Volume and structure of average total competitions in 5 years per level of categorisation.

Figure 4 presents the volume and structure of different categorisation levels. On average, NC judokas competed in 7 ± 1 competitions; PC judokas in 9 ± 1 competitions; IC judokas in 9 ± 2 competitions; WC judokas in 8 ± 0 competitions per year, with the majority of abroad competitions in an average of 5.47 competitions and an average of 2.73 home competitions per year.

Table 2 presents the dropout of judo athletes that stopped competing after 10 years. The Average dropout of all athletes is 52%, with the smallest dropout in the World class categorisation of 33% and the highest in the National class categorisation of 60%. 

Spearman correlation coefficient has shown a significant positive correlation of Competition Success as a sum of all points in 5 years (CS-5Y) with the number of competition categories (CC) in the year 2009 (*r* = 0.646; *p* = 0.001); 2010 (*r* = 0.491; *p* = 0.001) and 2011 (*r* = 0.302; *p* = 0.041). A significant negative correlation was noted between CS-5Y and CC in the year 2012 (*r* = −0.383; *p* = 0.009) and year 2013 (*r* = −0.436; *p* = 0.002). A significant positive correlation was noted between: CS in the year 2013 and Competition status (dropout) in 2018 (*r* = 0.369; *p* = 0.012); Competition status (dropout) in 2018 and number of abroad competitions in 2013 (*r* = −0.323; *p* = 0.029). Categorisation level has shown a positive correlation with the number of competitions abroad (*r* = 0.732; *p* = 0.001) and the total number of all competitions (*r* = 0.342; *p* = 0.020). The age of participants showed a significant negative correlation with categorisation level (*r* = −0.355; *p* = 0.015); the number of competitions abroad (*r* = −0.646; *p* = 0.001) and a total number of all competitions (*r* = −0.320; *p* = 0.030). 

Table 3 presents differences between categorisation groups for selected variables with the Kruskal–Wallis test (K-W). The K-W indicated a significant difference between groups in the number of competition categories in the years 2012 (H = 8.8, *p* ≤ 0.032, ES = 0.20 [large effect]) and 2013 (H = 10, *p* ≤ 0.019, ES = 0.22 [large effect]); however, the no significance was detected between groups after Bonferroni correction for multiple tests. There were also significant differences in the number of competitions between categorisation classes. K-W significant differences were also shown between Total Competitions at Home (H = 12.9, *p* ≤ 0.005, ES = 0.29 [large effect]) between WC and PC (*p* = 0.009) and Total Competitions Abroad (H = 24.7, *p* ≤ 0.000, ES = 0.55 [large effect]) between NC and PC (*p* = 0.023), NC and IC (*p* = 0.001) and NC and WC (*p* = 0.001). 

Table 4 presents differences between categorisation groups for selected variables with the K-W. The K-W indicated a significant difference in CS 2009 (H = 15.1, *p* ≤ 0.002, ES = 0.35 [large effect]) between NC and IC (*p* = 0.039), NC and WC (*p* = 0.010) and WC and PC (*p* = 0.037); CS 2010 (H = 15.9, *p* ≤ 0.002, ES = 0.37 [large effect]) between NC and IC (*p* = 0.013), NC and WC (*p* = 0.034) and PC and IC (*p* = 0.026); CS 2011 (H = 14.7, *p* ≤ 0.002, ES = 0.33 [large effect]) between NC and IC (*p* = 0.021), NC and WC (*p* = 0.015); CS 2012 (H = 16.1, *p* ≤ 0.001, ES = 0.36 [large effect]) between NC and WC (*p* = 0.003); in CS 2013 (H = 21.3, *p* ≤ 0.001, ES = 0.47 [large effect]) between NC and IC (*p* = 0.002), NC and WC (*p* = 0.002) and WC and PC (*p* = 0.042) and Total Competition Success in 5 Years (H = 22.2, *p* ≤ 0.001, ES = 0.49 [large effect]) between NC and IC (*p* = 0.001), NC and WC (*p* = 0.002) and WC and PC (*p* = 0.047).

## 4. Discussion

Results of the present study demonstrate that youth athletes, on average, compete in 8 ± 2 competitions per year. The structure of abroad competitions is greater for the WC, IC and PC categorised athletes, meaning NC athletes focus more on home competitions. On average, athletes compete in 6 ± 2 home and 2 ± 2 abroad competitions. Additionally, the data showed that youth athletes compete in 3 ± 1 age categories per year. This is also the first study in judo that reports youth judokas involvement in higher age categories and the so-called ‘’playing-up’’ problematic in youth sport. Additionally, a positive association was noted between 5 Years competition success and the number of age categories in years 2009, 2010 and 2011. On the contrary, in the final observed years, 2012 and 2013, the competitions success in 5 years showed a negative association with the number of age categories. Additionally, competition success in the final observed year 2013 showed a positive association with the dropout status in 2018 and a negative one with the number of abroad competitions in 2013. In total, 52% athletes dropout was noted in a 10 year period. 

From the current research, it is noted that the youngest athletes were in their primary U12 category, meaning they were under 12 years of age while competing in up to 3 higher age categories (U14, U16, U18). The recommended age limit to start training judo is 10 years [37]. The present study’s sample of judokas has undoubtedly started to train judo at an earlier age, as in Slovenia, judo is implemented as the concept of judo kindergarten for children aged from 4 to 6 years. Additionally, in primary school, judo can be chosen as an optional extra-curricular activity in the context of the so-called Little school of judo. As an example, In the Little school of judo in Ljubljana, in the school year 2011/2012, there were 1054 children actively involved in the training process. In the same academic year, four judo competitions were organised for them, with a sum of 1967 children competing and an average of 491.75 (±75.28) children per competition [38]. 

Early specialisation in sports is generally not recommended [7] based on the potential for injury [10,11] and as well as psychological burnout against the backdrop of a host of other potential sociological issues [39]. In addition, it can lead to dropout from sports because of lack of enjoyment, perceptions of competence, social pressures, competing priorities, pressure from the coaches, not getting along with coaches, anxiety and nervousness due to excessive criticism and physical factors like maturation and injuries [2,40]. Thus, it was highlighted that youth athletes who target high performance and results must engage in deliberate practice in their specialisation years to engage in tasks that will challenge their current performance [41]. Furthermore, it was reported that the road to expertise must constantly focus on improving weaknesses and producing successful outcomes by winning competitions [42]. Data from Figure 1 and Figure 2 show that the number of total competitions and achieved points is steadily increasing each year. This shows that categorised judokas are improving their weaknesses, challenging their performance, steadily producing better outcomes each year, and that their training programs are well planned and executed. In the current study, youth-categorised Slovenian judo athletes competed in 8 (±2) competitions per year. Additionally, differentiation between categorisation classes in the structure of home and abroad competitions was highlighted. In general, coaches need to include on average 8 competitions in their yearly periodisation plan. However, competitions abroad make periodisation even more difficult as they take more time to travel to and back. This especially implies the best judokas in the WC and IC, as it is known that travelling affects performance via numerous factors [43]. 

According to Barreiros and Fonseca [44] Portuguese judo athletes who started participating internationally at a senior level did not achieve the same high performance as their more successful counterparts. Present study data shows that Slovenian youth judokas start very early with the abroad competitions as some already start competing abroad in the U12 age category. Figure 1 reports that the higher the number of abroad competitions, the higher the competitive success regarding to achieved points, which gives abroad competitions an essential role in the process of gathering necessary experience for a young judoka. This is also supported by the negative correlation between the age of participants and the number of competitions abroad (*r* = −0.646; *p* =0.001), meaning that the younger judokas competed in more international competitions. Additionally, younger judokas competed more overall (*r* = −0.320; *p* = 0.030) and they also consequently achieved higher categorisation levels (*r* = −0.355; *p* = 0.015). Abroad competitions’ importance is also highlighted as the athletes with higher categorisation levels competed more abroad (*r* = 0.732; *p* = 0.001) and had in total, a higher number of overall competitions (*r* = 0.342; *p* = 0.020). Additionally, literature and elite coaches report that youth athletes competing abroad are exposed to all types of fights and that lack of abroad/international competitions may lead to insufficient competition experience, which greatly impacts performance at big competitions [45]. Therefore, the present study findings imply to an intensive early specialisation of youth judokas in Slovenia to achieve high sporting results in youth age groups. 

It was discussed that if we want more competent adult athletes, they should have better training conditions and spend more time practising and competing with better teammates and opponents in their youth [46]. This could be seen and supported in Figure 3 where data present NC and PC judokas started competing in 2009 at an average of 1.85 age categories. Competing in more-higher age categories has increased steadily every year, with 2010 1.96 age category, 2011 2.50 age category, 2012 2.99 age category and 2013 3.08 age category. So athletes are gathering more experience by fighting older and possibly better opponents in a short period. This is supported by present study data as Competition Success showed a positive association with the number of competition categories in the years 2009 (*r* = 0.646; *p* = 0.001); 2010 (*r* = 0.491; *p* = 0.001) and 2011 (*r* = 0.302; *p* = 0.041). Furthermore, the following shows that this approach from coaches is an effective way of boosting youth judokas competition success. However, the practice shows that youth judo athletes compete from 2 to 4 years older opponents in 1 to 2 higher age category competitions. Data alarmingly report that some youth judokas were also competing in 4 age categories, exposing them to competing with at least 6 years older opponents from seniors. We know there can be big differences in youth cognitive, physical, emotional and motivational capabilities within 1 year from the relative age effect [47], so we can imagine the differences when youth athletes compete with 2 to 4 years older opponents. In judo relative age effect was clearly identified in elite cadet and junior judo athletes [48]. When adolescent athletes are introduced to competitive events of the senior level, the stressors they encounter and how elite youth athletes cope with them should be understood and managed [49,50]. In the sports science literature, this phenomenon is known as ‘‘playing-up’’ when athletes train and compete with older peers [51]. To the best of our knowledge, this is the first study that would identify and put in context the so-called ‘‘playing-up’’ problematic/topic in judo and could also be terminologically adapted to be more judo-specific to ‘‘competing-up.’’ This topic has been briefly addressed in recent years in youth football [52,53] where playing-up has shown positive implications for performance and developmental outcomes in youth football. These findings are in line with our current data in judo. However, the football studies did not explore its association with the possible dropout and performance levels in later years. Nonetheless, the studies have highlighted some important findings where youth athletes playing-up struggled with the intensity of training and competitions and to fit in socially with older peers [53]. However, it was also recomemnded that those younger atheletes had better chances to integrate socially within older competitors when teammates introduced themselves and acively included youth copetitors in sport and their social activities [53]. In practical terms, that would mean that both the competing-up athlete and the older group competitors must be prepared to join or have youth athletes join-in their group. Therefore, coaches play an essential part in preparing both groups and laying the foundations for a smooth transition.

When youth athletes are exposed to numerous hours of deliberate practice without understanding the training context and having a precise aim will not lead to the desired effects in training or competitions [54]. However, the maturation effect on growth and physical performance in young judokas has been highlighted as it seems more relevant than the age effect [55,56]. Additionally, it has been shown that maturation attenuated the age effect and significantly affected upper body and handgrip strength in youth judokas [57]. This phenomenon could explain why we have so many youth judo athletes competing in higher age categories and being successful in them. 

Data present that coaches are trying to gain extra experience with their athletes by competing in higher age categories and accumulating an extensive competitive experience with older contestants as fast as possible. Some of them are too concentrated on quick results, which is often associated with their job requirements and elite youth result, which brings possible extra funding to the club or qualifying for an Olympic scholarship program. However, there are no shortcuts to physiological, psychological, technical-tactical and social components of youth athlete development. The coaches should not be short-sighted and focused on the early elite result. Some researchers state that you have to be among the best youth athletes if you want to be a top judoka later in the senior level [44]. However, on the contrary, only 7% of the male and 5% of the female judokas had maintained their competitive levels [17]. Similar findings have been found in Slovenia, where a significant dropout rate was found in athletes achieving top results in junior categories after transitioning to the senior category [58]. The same research reports that 49% of top athletes achieving excellent results in seniors had not been in the top earlier in the junior age category. Moreover, it was reported that 30% of athletes that had their best result at senior-level competitions failed to obtain the categorisation of perspective class when they competed in the junior category [58]. 

Therefore, successful competitive performance in early judo competition is not associated with success later in adulthood. We can observe that among the Athens 2004 Olympians, 56% made their first international appearance in the senior age category at the age 22.0 (±3.1 years) [59]. Additionally, the present study data showed that competitive success in 5 years was negatively associated with the number of competition categories in the year 2012 (*r* = −0.383; *p* = 0.009) and year 2013 (*r* = −.436; *p* = 0.002). This could imply that the early specialisation and accumulation of competitive performance via competitions in higher age categories is successful in a short period of time. Afterwards, it can have a negative impact on youth athelets and their competitor status. Athletes who were not performing at the highest level and had a high competitive success were also associated with a higher drop out in the year 2018 (*r* = 0.369; *p* = 0.012) and a lower number of abroad competitions (*r* = −0.323; *p* = 0.029). Nonetheless, coaches of elite youth judo athletes should try to focus on competitions abroad and quality training with older age groups to increase the quality and lower the possibility of athletes’ burnout and injuries. Additionally, specialisation strategies from Japan could be adopted where specialisation in female judokas starts at an average age of 14.2 years, while Japanese male athletes start specialised trainings at an average age of 16.3 years [11]. 

From the competitive volume and linear increase in competitive success presented in Figure 2, it can be summarised that the selection process and the training programs in younger age categories are well planned and executed for a good youth result. It was also presented that the Judo Federation of Slovenia had a positive trend and systematically achieved superior results over 2008–2013, both in junior and senior competitions [58]. Nonetheless, the current study showed that this came at a hefty price, with a 52% dropout of youth judokas over the next 10 years. The current analysis also showed that a wider view of the success analysis in sports, especially youth sports, needs to be considered. In this way, the initial research [58], combined with this analysis, could highlight the success and reveal the dropout problem more efficiently. Possibly, this approach could be an organisational effect of smaller countries like Slovenia (with a population of 2.106.215 [60]), which do not have a large pool of competitors and need to rely on a small number of highly talented individuals and hope they do not get injured or drop out for any other reason. This phenomenon of smaller countries’ organisations and success in judo needs further research to give us greater insight. The home and abroad competitions structure highlights the importance of abroad competitions in developing youth judo athletes. Therefore, coaches should plan that abroad competitions are attended at an early age in order to achieve elite youth results and have a good foundation for the senior age category. Research has shown [44] that male judokas participating in major events started competing sooner at an international level (U = 0.266, *p* = 0.007). Nevertheless, it is recommended that competitions should be implemented inside the primary age category or no more than one age category higher, as this would ensure a systematic development and progressive load of a young body. Judo has been identified as a sport of slow technical maturation and it requires adaptation before its mastery in competition [3]. Therefore, coaches should include more competitions abroad in competitors’ primary age groups in their yearly periodisation. Additionally, elite judo coaches highlighted and recommended that competition in training should be widely implemented because it helps in the development of the motor-perceptual, conditional, technical, tactical and psychological potential of judokas [61]. Additionally, training sessions with higher age groups would be a good solution for gathering additional experience in a more controlled environment. Peak performance can also be achieved by exploring various sports in childhood and combining 4000 to 6000 h of deliberate practice in a chosen sport [25]. Early diversification in sports was suggested [62], as it might stimulate physiological and cognitive adaptations, which lay the groundwork for specialised and cognitive capacities necessary for later expertise [63]. Furthermore, fewer hours of training and high health satisfaction characterise low-risk groups for dropping out from sports for elite youth athletes [29], which would, in short translate as “train smarter, not harder”.

The current study needs to acknowledge some limitations. The main one is that there was no possibility to identify possible injuries and their impact on competing in higher age categories. Additionally, the reason for the athletes’ dropout is unknown. Therefore, further mixed-methods studies are recommended to get the dropout athletes’ feedback on how competing in multiple higher age categories impacted them and what later led to their drop out from judo and competitions. Additionally, coaches’ feedback and opinion on competing in higher age categories as a tool to fast-track combat experience should be further researched. Finally, the starting age when the athletes started with judo and later with more intensive training was not known as this would give us a better understanding of the early specialisation.

## 5. Conclusions

Youth judokas are regularly competing in domestic or international competitions. Throughout the years, they gain experience in different patterns. There is a tendency to accumulate youth judoka fighting experience as much as possible and as fast as possible. This is done by competing in more-higher age categories, resulting in youth judokas competing with at least 2 to 6 or more years’ older opponents in at least 1 to 3 higher age categories. Additionally, competing abroad significantly affects fighting experience and, consequently, greater competitive success. This approach was shown to be successful in creating youth medalists. However, just as a short-term solution/plan, if implemented for too long, it starts to negatively affect competition success and increases youth athletes’ dropout. Therefore, coaches’ main focus should be to prepare youth judokas on the senior level through the system of national and international competitions within their age category. They should include more competitions abroad in competitors’ primary age group, while training sessions could be done with higher age groups which would allow for gathering additional experience in a more controlled environment in their yearly periodisation. This would ensure adequate muscle-skeletal development of youth judo competitors and steady progress and development of required technical-tactical skills for competing at the senior level. 

## Figures and Tables

**Table 1:** Descriptive statistics of the sample.

Variables	Mean ± SD
Age	19.5 ± 2.4
Sex (%)	
Female	47.8
Male	52.2
Categorisation (%)	
1—World Class	6.5
2—International Class	10.9
3—Perspective Class	50.0
4—National Class	32.6
2009 Starting age category National Class	6 ± 1
2009 Starting age category Perspective Class	6 ± 1
2009 Starting age category International Class	3 ± 0.5
2009 Starting age category World Class	3 ± 0
2009 Average Competition category	5 ± 2
U21 participants in 2009 (n)	11
U18 participants in 2009 (n)	8
U14 participants in 2009 (n)	17
U12 participants in 2009 (n)	10
Total Home Competitions in 5 years	30 ± 10
Total Abroad Competitions in 5 years	10 ± 8
Home Competitions per year	6 ± 2
Abroad Competitions per year	2 ± 2
Total Competitions per Year	8 ± 2
CS—Points 2009 (n = 44)	682 ± 843
CS—Points 2010 (n = 44)	941 ± 1324
CS—Points 2011 (n = 46)	1038 ± 1286
CS—Points 2012 (n = 46)	1251 ± 1291
CS—Points 2013 (n = 46)	1753 ± 1550
Competition categories per year	3 ± 1

Legend: 7 age competition categories 7-U12, 6-U14, 5-U16, 4-U18, 3-U21, 2-U23, 1-Seniors; CS—competition success.

**Table 2:** Dropout of athletes in 10 years.

VARIABLES	Competing in 2018		Dropout
Categorisation Level	YES	NO	%
National	6	9	60
Perspective	11	12	52
International	3	2	40
World Class	2	1	33
TOTAL	22	24	52

**Table 3:** Difference between Categorisation groups for the number of competition categories and competition distribution in selected years with Kruskal–Wallis test and effect size.

Variable	Group	Mean	SD	Mean Rank	χ^2^	Sig	ES
C CAT 2009	WC	2	1	31.50	5.73	0.126	0.133
	IC			31.70			
	PC			19.91			
	NC			21.36			
C CAT 2010	WC	2	1	26.50	5.77	0.123	0.134
	IC			31.00			
	PC			18.52			
	NC			25.35			
C CAT 2011	WC	2	1	12.50	2.47	0.482	0.055
	IC			23.60			
	PC			24.24			
	NC			24.53			
C CAT 2012	WC	3	1	9.50	8.79	0.032 ^#^	0.195
	IC			12.90			
	PC			26.48			
	NC			25.27			
C CAT 2013	WC	3	1	7.17	9.99	0.019 ^#^	0.222
	IC			13.00			
	PC			26.39			
	NC			25.83			
T C H	WC	30	10	2.33	12.89	0.005 ^†^	0.286
	IC			14.20			
	PC			28.33			
	NC			23.43			
T C A	WC	10	8	44.67	24.67	0.000 ^‡,₸,×^	0.548
	IC			38.20			
	PC			24.98			
	NC			12.10			
T C 5Y	WC	40	12	25.33	6.67	0.083	0.148
	IC			28.80			
	PC			26.85			
	NC			16.23			

Legend: ES—effect size; C—competition; CAT—category; T—total; H—home; A—abroad; 5Y—in 5 years; NC—National class; PC—Perspective class; IC—International Class; WC—World class; ^#^—no significance between groups after Bonferroni correction for multiple tests; ^†^—sig. difference between WC and PC; ^‡^—sig. difference between NC and PC; ^₸^—sig. difference between NC and IC; ^˟^—sig. difference between NC and WC.

**Table 4:** Difference between Categorisation groups for Competition Success (CS) for selected years with Kruskal–Wallis test and effect size.

Variable	Group	Mean	SD	Mean Rank	χ^2^	Sig	ES
CS 2009	WC	682	843	42.33	15.10	0.002 ^₸,×,†^	0.351
	IC			34.90			
	PC			20.66			
	NC			16.71			
CS 2010	WC	941	1324	40.00	15.93	0.002 ^₸,×,‖^	0.370
	IC			37.90			
	PC			19.85			
	NC			17.23			
CS 2011	WC	1038	1286	41.33	14.68	0.002 ^₸,×^	0.326
	IC			35.90			
	PC			23.59			
	NC			15.67			
CS 2012	WC	1251	1291	44.00	16.13	0.001 ^×^	0.358
	IC			30.50			
	PC			25.41			
	NC			14.13			
CS 2013	WC	1753	1550	45.00	21.25	0.001 ^₸,×,†^	0.472
	IC			39.70			
	PC			22.80			
	NC			14.87			
T CS 5Y	WC	5594	5413	45.00	22.18	0.001 ^₸,×,†^	0.493
	IC			39.90			
	PC			23.11			
	NC			14.33			

Legend: ES—effect size; CS—competition success (points); CAT—category; T—total; 5Y—in 5 years; NC—National class; PC—Perspective class; IC—International Class; WC—World class; ^†^—sig. difference between WC and PC; ^₸^—sig. difference between NC and IC; ^˟^—sig. difference between NC and WC; ^‖^—sig. difference between PC and IC.

## Data Availability

The raw data supporting the conclusions of this article will be made available by the author upon reasonable request to any qualified researcher.
